# Age and cellular context influence rectal prolapse formation in mice with caecal wall colorectal cancer xenografts

**DOI:** 10.18632/oncotarget.12312

**Published:** 2016-09-28

**Authors:** Joke Tommelein, Félix Gremonprez, Laurine Verset, Elly De Vlieghere, Glenn Wagemans, Christian Gespach, Tom Boterberg, Pieter Demetter, Wim Ceelen, Marc Bracke, Olivier De Wever

**Affiliations:** ^1^ Laboratory of Experimental Cancer Research, Department of Radiation Oncology and Experimental Cancer Research, Ghent University, Ghent, Belgium; ^2^ Cancer Research Institute Ghent (CRIG), Ghent, Belgium; ^3^ Department of Surgery, Ghent University Hospital, Ghent, Belgium; ^4^ Department of Pathology, Erasme University Hospital, Université Libre de Bruxelles, Brussels, Belgium; ^5^ Institut National de la Santé et de la Recherche Médicale, INSERM, Department of Molecular and Clinical Oncology, Université Paris VI Pierre et Marie Curie, Paris, France

**Keywords:** rectal prolapse, COLO320DM, colorectal cancer, orthotopic, mouse model

## Abstract

In patients with rectal prolapse is the prevalence of colorectal cancer increased, suggesting that a colorectal tumor may induce rectal prolapse. Establishment of tumor xenografts in immunodeficient mice after orthotopic inoculations of human colorectal cancer cells into the caecal wall is a widely used approach for the study of human colorectal cancer progression and preclinical evaluation of therapeutics. Remarkably, 70% of young mice carrying a COLO320DM caecal tumor showed symptoms of intussusception of the large bowel associated with intestinal lumen obstruction and rectal prolapse. The quantity of the COLO320DM bioluminescent signal of the first three weeks post-inoculation predicts prolapse in young mice. Rectal prolapse was not observed in adult mice carrying a COLO320DM caecal tumor or young mice carrying a HT29 caecal tumor. In contrast to HT29 tumors, which showed local invasion and metastasis, COLO320DM tumors demonstrated a non-invasive tumor with pushing borders without presence of metastasis. In conclusion, rectal prolapse can be linked to a non-invasive, space-occupying COLO320DM tumor in the gastrointestinal tract of young immunodeficient mice. These data reveal a model that can clarify the association of patients showing rectal prolapse with colorectal cancer.

## INTRODUCTION

Rectal prolapse is the complete protrusion of the rectum through the anal canal. In children, rectal prolapse is related to chronic constipation, acute diarrheal disease, cystic fibrosis, and neurologic/anatomic abnormalities [1]. In adults, rectal prolapse is associated with pregnancy, obesity, perineal injury, chronic constipation, or other disorders leading to enhanced intra-abdominal pressure [2]. Interestingly, rectal prolapse can also occur as a symptom of colorectal cancer (CRC). Patients with rectal prolapse are reported to have a 4.2-fold relative risk for CRC in comparison with a control group [3]. This may indicate that rectal prolapse is induced by CRC. Here, we demonstrate a murine model that resembles the situation of these patients.

To study CRC *in vivo*, several murine models are available [4]. Genetically engineered mouse models are induced by overexpression or knockdown of a given dominant oncogene/tumor suppressor gene, respectively. Of note, this approach is not representative of the natural history of the neoplasia since malignant human tumors are considered to be induced by the sequential combination and progressive accumulation of 6-9 genetic defects. Multiplexed genome engineering by transfection-based CRISPR/Cas9 delivery may offer novel opportunities to study the complexity of cancer [5].

Direct orthotopic implantation of human CRC cells in their natural microenvironment is actually considered as a pertinent approach for modeling CRC progression. High tumor take rates and spontaneous metastases at clinically relevant rates were observed when HT29, a human CRC cell line, was injected orthotopically into the caecal wall of immunodeficient mice [6]. Furthermore, the orthotopic model is shown to be a relevant model for therapy studies [6–8]. Intra-caecal inoculation of several cell lines such as HCT-116, SW-620, DLD-1 (HCT8/E11), HT29, KM12 has been reported [6–13].

COLO320DM cells differ from other reported epithelial cell lines such as HCT8/E11, HT29, SW-480 and HCT-116. Most of the CRC cell lines adhere strongly to the culture flask and form dense cell colonies. They have cell membranes with interdigitating brush borders and secrete variable amounts of carcinoembryonic antigen. COLO320DM does not form dense cell colonies and the cells easily detach from the culture flask. The cells are round with few microvilli or desmosomes. COLO320DM is reported to secrete serotonin and catecholamines such as norepinephrine and epinephrine and are referred to as amine precursor uptake and decarboxylation (APUD) cells [14]. Moreover, COLO320DM contains double minutes, defined as extrachromosomal DNA [14]. Intra-caecal injection of single cell suspensions of COLO320DM was not yet described.

Because the multifactorial interactions between cancer cells and the tumor stroma determine growth, angiogenesis and metastasis to distal organs, the orthotopic model is the most realistic replication of CRC [6, 15]. The tumor stroma consists of ostensibly normal cells such as immune cells, endothelial cells and cancer-associated fibroblasts (CAFs) as principal components. We have previously shown that colorectal CAFs contribute to most of the cancer hallmarks, promoting CRC progression and metastasis [16].

In the present study, young mice, in contrast to adult mice, were susceptible to rectal prolapse when a growing caecal tumor of COLO320DM cells was present. However, rectal prolapse was not observed in adult mice carrying a COLO320DM caecal tumor or young mice carrying a HT29 caecal tumor. To our knowledge, we are the first to describe an association between orthotopic intra-caecal xenografts of CRC cells and rectal prolapse in mice.

## RESULTS

### COLO320DM characterization and subcutaneous (SC) implantation

**Figure 1:**
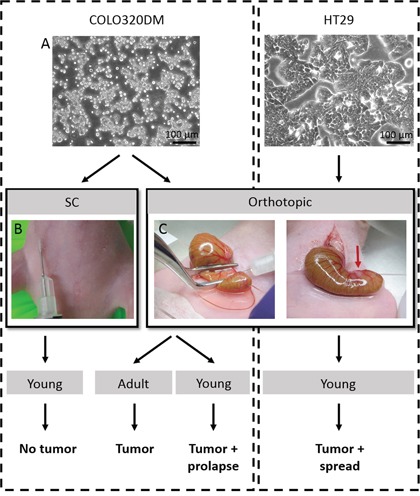
Overview of the performed *in vivo* experiments **A.** Phase/contrast pictures of human CRC cell lines COLO320DM and HT29. **B.** Picture of SC inoculation of COLO320DM. **C.** In the orthotopic model, the caecum was gently exteriorized and flattened with a forceps before injecting the cells into the caecal wall. A small bleb was a sign of successful injection. SC = subcutaneous.

**Figure 2:**
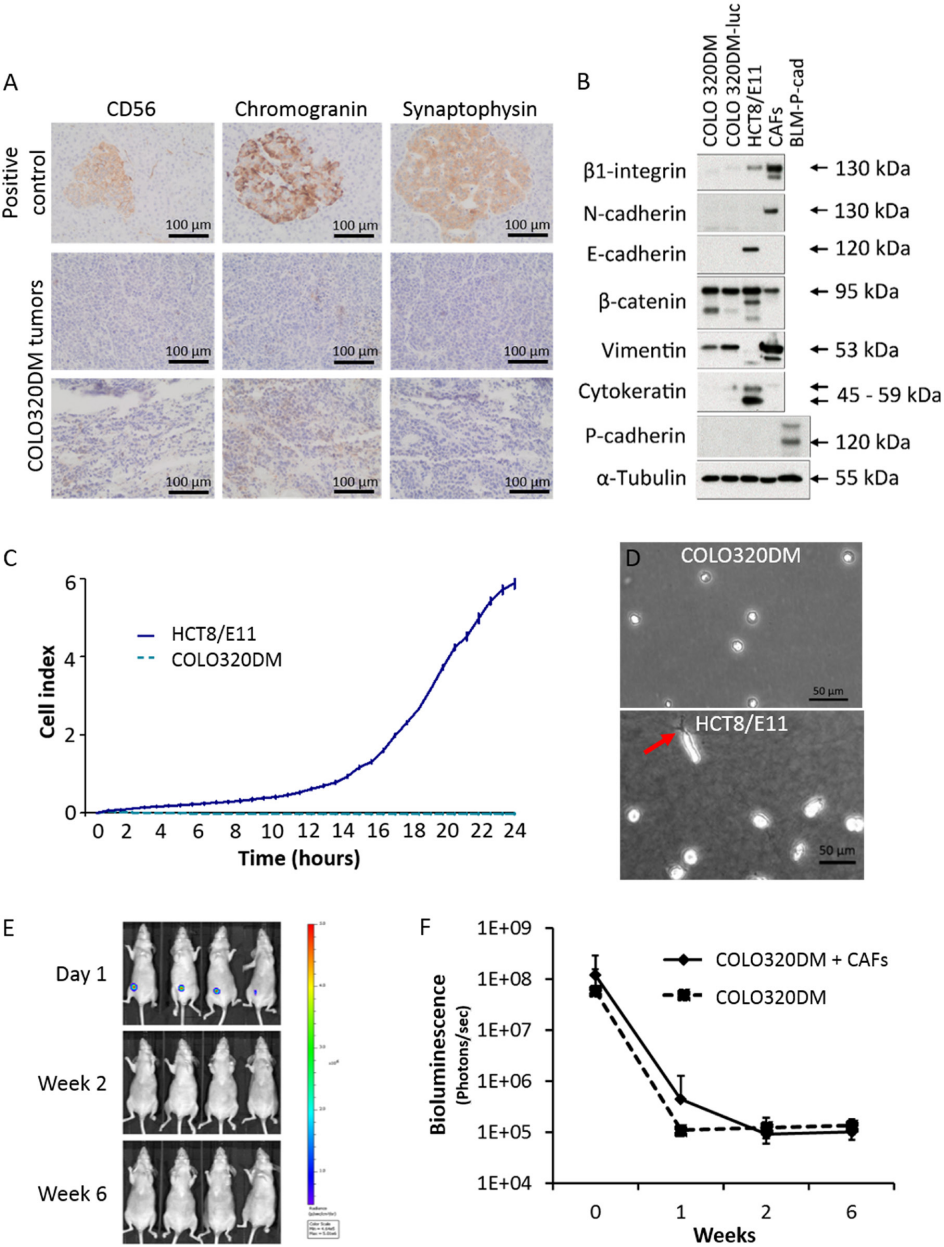
COLO320DM characterization and SC implantation **A.** Immunohistochemistry for neuroendocrine markers CD56, chromogranin and synaptophysin in COLO320DM tumors. Normal pancreas was used as positive control. **B.** Western blot for different EMT markers in COLO320DM and luciferase transfected COLO320DM cells compared to HCT8/E11 and CAFs. **C.** Real-time monitoring of CRC cell migration by measuring electrical impedance during 24h. The cell index is displayed as mean ± SD. **D.** Phase/contrast image of COLO320DM and HCT8/E11 cells on type I collagen gels. The arrow indicates an invasive extension. **E.** *In vivo* bioluminescence monitoring of young mice subcutaneously inoculated with COLO320DM cells in combination with CAFs. Four representative mice are shown. **F.** Bioluminescence quantification of young mice subcutaneously inoculated, presented as the mean bioluminescence ± SD of mice inoculated with COLO320DM alone or in combination with CAFs.

*In vitro* cultures of COLO320DM showed mainly a round morphotype (Figure 1A). Neuroendocrine origin of the cell line was suggested by Quinn et al. [14] but neuroendocrine markers CD56, chromogranin and synaptophysin were negative in COLO320DM tumors (Figure 2A). For a neuroendocrine tumor two out of three markers should be positive. Lack of expression of cytokeratin, an intermediate filament protein present in the intracytoplasmic cytoskeleton of epithelial cells, confirmed a non-epithelial phenotype. Furthermore vimentin, an intermediate filament expressed in mesenchymal cells, was detected in COLO320DM. Membrane receptor β1-integrin and type-1 transmembrane proteins N-cadherin, E-cadherin and P-cadherin, which are important for cell-cell and cell-matrix adhesion, were not expressed in COLO320DM. Accordingly, this cell line had no tight cell-cell contacts despite the expression of β-catenin, which needs cadherins to form an association with the actin filaments (Figure 2B). In contrast to epithelial HCT8/E11 cells, COLO320DM cells did not show serum-induced chemotactic migration as demonstrated by real-time monitoring of cell migration (Figure 2C). Type I collagen invasion assay revealed a non-invasive morphotype of COLO320DM cells, while in HCT8/E11 cells some invasive extensions were observed (Figure 2D). Previously, two studies showed tumor development following SC inoculation of COLO320DM in BALB/c nu/nu mice [17, 18]. In a first experiment Swiss nu/nu mice were SC injected with COLO320DM either alone or in combination with CAFs (Figure 1B). Tumor growth was monitored by bioluminescence imaging (Figure 2E, 2F). One day after SC injection, all mice (n=15) demonstrated a luciferase positive signal at the site of injection. This indicates that the procedure was well performed and cancer cells were present initially. However, one week later the signal had disappeared and remained absent up to 6 weeks post-inoculation (Figure 2E, 2F). In accordance, no macroscopic tumor was detectable. Histological assessment confirmed that COLO320DM cells injected at the SC, ectopic site did not develop tumors. Results were similar for mice injected with COLO320DM alone or in combination with CAFs (Figure 2F).

### Orthothopic implantation of COLO320DM or HT29

To resemble a more natural environment for CRC, an orthotopic model was developed by intra-caecal injection of CRC cells in combination with CAFs. Under 2.5x magnification and using a 30G needle, cells were injected in the caecal wall. Initially, the injection technique was established using methylene blue injections. When injected too deeply, the dye was diluted by the bowel content. A too superficial injection resulted in peritoneal leakage (data not shown). Bleb-formation after injection served as a quality control for the technique (Figure 1C). Orthotopic injection turned out to be a safe procedure as no animals showed morbidity or mortality because of the surgery. This procedure was performed with COLO320DM in adult and young mice and with HT29 cells in young mice. CRC cells were combined with CAFs in every experiment. In contrast to SC injection, orthotopically injected mice were able to develop a tumor. In all groups a tumor take rate of 62.5% was observed, suggesting the reproducibility of the technique.

### Orthotopic injection of COLO320DM: tumor growth and survival of adult versus young mice

**Figure 3:**
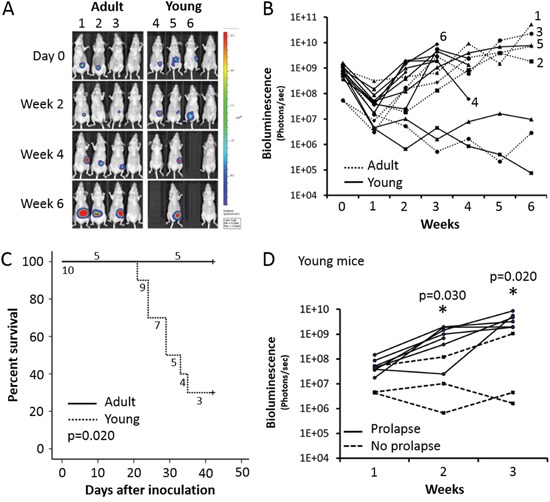
Adult versus young mice orthotopically injected with COLO320DM **A.** *In vivo* bioluminescence monitoring. Four representative adult or young mice are shown. **B.** Quantification of bioluminescence. Only mice carrying a COLO320DM caecum tumor are included. **C.** Survival curve of mice carrying a COLO320DM caecum tumor. **D.** Bioluminescence quantification of young mice with a COLO320DM caecal tumor the first three weeks after inoculation.

**Figure 4:**
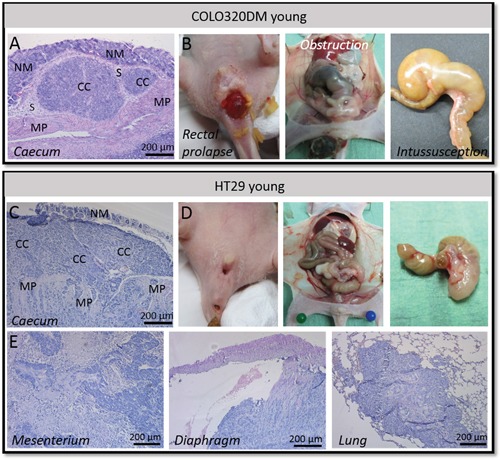
Orthotopic COLO320DM versus HT29 tumors in young mice **A.** H&E staining of the local COLO320DM tumor showing pushing borders. **B.** Rectal prolapse, obstruction of the large bowel and intussusception of the caecum was observed in mice carrying a COLO320DM tumor. **C.** H&E staining of the primary HT29 tumor demonstrating infiltration in the normal adjacent host tissue. **D.** No rectal prolapse, obstruction of the large bowel or intussusception of the caecum was observed using HT29 cells. **E.** H&E staining of HT29 cancer cell spread to mesenterium, diaphragm and lung. NM = normal mucosa, CC = cancer cells, S = submucosa, MP = muscularis propria.

Weekly bioluminescence imaging was used to follow up mice orthotopically injected with COLO320DM and CAFs. Results for four representative adult and young mice are displayed in Figure 3A. A local tumor was observed in five out of eight (62.5%) adult mice and in ten out of sixteen (62.5%) young mice (Table 1). Quantification of bioluminescence revealed no significant changes in adult compared to young mice (Figure 3B). However, young mice with a caecum tumor showed a significant lower survival than adult mice with a caecum tumor (p=0.020) (Figure 3C). Seven out of ten (70%) young mice with a caecum tumor showed rectal prolapse and had to be sacrificed at the latest six weeks after inoculation (Figure 4B). In contrast, no rectal prolapse occurred in adult mice (Table 1). Consequently, young mice had a significantly higher chance to develop rectal prolapse (p=0.026). Rectal prolapse was not observed in young mice that did not grow a local tumor after intra-caecal injection with COLO320DM (p=0.011). In the first three weeks after inoculation of the cells, a correlation is observed between the intensity of the bioluminescent signal and the occurrence of prolapse in the six weeks following the inoculation (p=0.005). Young mice with a higher bioluminescent signal two and three weeks post-inoculation had a higher chance to develop rectal prolapse (p=0.030 and 0.020 respectively, Figure 3D).

**Table 1:** Local tumor growth and appearance of rectal prolapse in young or adult mice SC or orthotopically injected with human CRC cell lines

Location of injection	Cell line	Age	Mice (n)	Local tumor take rate (%)	Rectal prolapse (%)
SC	COLO320DM	6 weeks	15	0/15 (0 %)	-
Intra-caecal	COLO320DM	6 weeks	16	10/16 (62.5 %)	7/10 (70 %)
Intra-caecal	COLO320DM	> 12 weeks	8	5/8 (62.5 %)	0/5 (0 %)
Intra-caecal	HT29	6 weeks	8	5/8 (62.5 %)	0/5 (0 %)

SC = subcutaneous.

### Necropsy and histopathology of mice exhibiting rectal prolapse

When an animal started to exhibit symptoms of morbidity it was sacrificed and necropsied followed by histological examination. Young mice orthotopically injected with COLO320DM exhibiting rectal prolapse were characterized by a protrusion of the rectum beyond the anus. Necropsy of these mice demonstrated intussusception of the caecum, meaning that the caecum invaginated the large bowel. This intussusception resulted in bowel obstruction (Figure 4B). Hematoxylin and eosin (H&E) staining of primary COLO320DM tumors showed a non-invasive tumor with pushing borders. The tumor was located in the submucosa (Figure 4A). Also other organs were prelevated, but H&E stainings could not reveal metastases confirming the local intra-abdominal bioluminescent signal.

### Orthotopic injection in young mice: HT29 versus COLO320DM

HT29 has a more epithelial morphotype with tight cell-cell contacts and is more differentiated than COLO320DM (Figure 1A). Five out of eight (62.5%) young mice orthotopically injected with HT29 and CAFs developed a local tumor. In contrast to young mice orthotopically injected with COLO320DM, no rectal prolapses were observed (p=0.026)(Table 1). The caecum implanted with HT29 and CAFs did not invaginate the large bowel and no obstruction was seen in the first six weeks after inoculation. H&E staining of the primary tumor showed differentiated cancer cell nests that invaded into the normal adjacent host tissue. HT29 gave rise to spread of cancer cells to several sites such as mesenterium, liver, diaphragm and lungs (Figure 4E). This systemic spread did not cause death in the first six weeks post-inoculation. Survival of young mice with a local HT29 tumor was therefore significantly better than for young mice with a COLO320DM tumor (p=0.020; Supplementary Figure S1).

## DISCUSSION

The orthotopic model yields a tumor take rate of 62.5% in all groups, independent of the cell line used or the age of the mice. In literature tumor take rates from > 50% up to 100% are described for different epithelial cell lines, such as HCT-116, SW-620, DLD-1 or HCT-8/E11 and HT29 (Table 2). Although these cell lines were also intra-caecally injected in immunodeficient mice aged from 4 weeks until 12 weeks, no rectal prolapse was reported (Table 2) [6–13]. This is in line with our findings that rectal prolapse only occurred in young mice bearing a COLO320DM caecum tumor and not in young mice with an epithelial HT29 caecum tumor in the same mouse strain from the same age (Table 2). Consequently, we hypothesize that the development of rectal prolapse is due to a space-occupying, non-invasive tumor or through the intrinsic characteristics of the COLO320DM cell line either through secretion of serotonin or through its unique response to a young environment. The presence of a caecum tumor was provocative to rectal prolapse, excluding that the technical procedure was the cause.

**Table 2:** Overview of the literature reporting intra-caecal injection of single cell suspensions and comparison with our results

Author	Mouse strain	Gender	Age	Cell line	Cell number	Take rate	Prolapse
Our results	Swiss Nu/Nu	female	6 weeks	COLO320DM	1×10^6^	62.5%	Yes
			> 12 weeks	COLO320DM	1×10^6^	62.5%	No
			6 weeks	HT29	1×10^6^	62.5%	No
Céspedes et al. [9]	Swiss Nu/Nu	male	4 weeks	HCT-116	2×10^6^	75%	No
				SW-620	2×10^6^	75%	No
				DLD-1	2×10^6^	88%	No
Hackl et al. [6]	CB17SCID	female	6 weeks	HT29	5×10^5^	87.5 – 100%	No
				HCT-116	5×10^5^	87.5 – 100%	No
Van Hoorde et al. [10]	Swiss Nu/Nu	male	6 weeks	HCT-8/E11	?	> 50 %	No
Sasaki et al. [7]	Athymic nude (NCl-nu)	male	8-12 weeks	SW-620	5×10^5^	100%	No
Kitadai et al. [11]	Athymic Ncr-nu/nu	male	8-12 weeks	KM12SM	2×10^6^	100%	No
Rebhun et al. [12]	Athymic nude (Ncl-nu)	male	8-12 weeks	HT29	1×10^6^	100%	No
Yokoi et al. [8]	Athymic nude (Ncl-nu)	male	8-12 weeks	HT29	1×10^6^	100%	No
Morikawa et al. [13]	Athymic BALB/c nude	male	8 weeks	KM12	1×10^6^	100%	No

COLO320DM cells are referred to as APUD cells secreting serotonin and catecholamines such as norepinephrine and epinephrine [14], but they do not express the neuroendocrine markers CD56, chromogranin or synaptophysin. In comparison with epithelial HCT8/E11, COLO320DM cells exhibit epithelial to mesenchymal transition (EMT) features demonstrated by the lack of epithelial markers E-cadherin, EpCAM, cytokeratin and the expression of mesenchymal marker vimentin [19, 20]. Nevertheless, the presence of these EMT markers does not correspond with a pro-migratory/invasive phenotype *in vitro* nor *in vivo*.

Serotonin (5-hydroxytryptamine, 5-HT) regulates the colonic transit by modulating intestinal motility, sensitivity, inflammation and secretion [21–23]. Mast cells are also a source of serotonin and the number of mast cells is increased in adults with irritable bowel syndrome (IBS) [22]. Similar results were obtained by Cremon et al. who demonstrated a 10-fold higher serotonin release in patients with IBS [24]. In accordance, children with IBS showed a higher serotonin content in the rectal mucosa [25]. These examples prove that changes in serotonin availability can disturb the balance in the gastrointestinal tract. Serotonin can influence the immune system and serotonin synthesis is increased in Crohn's disease [26]. Also in experimental models of colitis the serotonin content was increased [27, 28]. Treating serotonin-lacking mice with 5-HTP, the direct precursor of serotonin, increased the severity of colitis demonstrating a pivotal mediating role of serotonin in intestinal inflammation [29, 30]. Rectal prolapse frequently occurred in mice with rectal inflammatory bowel disease [31]. Consequently, serotonin may play a role in the development of rectal prolapse. However, Dolk *et al.* immunocytochemically investigated the occurrence and distribution of endocrine cells secreting serotonin on patients with rectal prolapse and on normal rectal mucosa and did not detect significant differences [32].

Rectal prolapse, which is also known as procidentia, is defined as a protrusion of all layers of the rectal wall through the anal canal [33–36]. This corresponds to complete or full-thickness rectal prolapse. If the rectal wall prolapses but does not protrude through the anus, it is termed an occult (internal) rectal prolapse or a rectal intussusception [36–38]. The prevalence of rectal prolapse in patients is low and occurs at the extremes of age [35, 36, 39]. In children it is usually diagnosed by the age of 3 years [36, 39]. This is in accordance to our results which demonstrated rectal prolapse in young mice. In adults, rectal prolapse mainly occurs in patients over 50 years old and women are more affected, representing 80% to 90% of the patients [35, 36, 39]. Elderly females are more susceptible because of the damaging effect of pregnancy and menopause on the pelvic floor [40]. Since the mice exhibiting prolapse were young and nulliparous, this cannot be a cause in our experiments. In children, rectal prolapse may result from the lack of the natural sacral curve. Because there is little or no anorectal angulation any increase in intra-abdominal pressure can lead to an increase in anorectal pressure, causing rectal prolapse [41].

In contrast to humans, rectal prolapse occurs regularly in laboratory mice. The higher incidence in mice may be explained by the short and poorly supported distal colon of mice, which lacks a serosal covering [42, 43].

A space-occupying tumor within the gastrointestinal tract may increase intra-abdominal pressure and can be a predisposing factor for rectal prolapse [42]. In our study, the tumor derived from COLO320DM orthotopic injection initiated intussusception in young mice, which then resulted in rectal prolapse because of obstruction of the bowel and abdominal pressure. In accordance, several case studies reported rectal prolapse when adenocarcinoma of the sigmoid colon was present [44–46]. Patients with rectal prolapse showed a 4.2-fold relative risk for CRC in comparison with a control group [3]. In line with this data, McNicol et al. report that the association of rectal prolapse and rectal adenocarcinoma is not that rare. In one year two cases of complete rectal prolapse resulted in the diagnosis of primary sigmoid or rectal adenoacarcinoma [47]. Therefore, tumor masses may be associated with the development of intussusception. H&E staining of orthotopic COLO320DM tumors demonstrated a tumor mass with pushing borders, but without any invasive characteristics. Our data suggests that bioluminescent signal of the first three weeks post-inoculation may predict prolapse in the six weeks following orthotopic inoculation with COLO320DM in young mice. Bioluminescent signal corresponds with the number of cancer cells, thus a larger tumor in the beginning was associated with rectal prolapse later on.

COLO320DM cells may uniquely react to a young environment. To simulate an aging environment we induced replicative senescence in CAFs and investigated the functional impact of its secretome on COLO320DM cell growth. Supplementary Figure S2 shows young and replicative-aged CAFs differentially characterized by morphology, β-galactosidase activity and increased γ-H2AX and p21cip1 staining (Supplementary Figure S2A-S2C). The secretome of replicative aged CAFs reduced cancer cell growth compared to the secretome of young CAFs (p=0.021; Supplementary Figure S2D). CAFs play a critical role in CRC progression, particularly by the secretion of several factors [16]. The secreted factors from CAFs may be age-dependent as was shown by Kaur et al. for dermal fibroblasts [48]. Aged fibroblasts secreted more sFRP, an inhibitor of β-catenin, leading to decreased melanoma cell proliferation and increased invasion [48]. However, the reduction of cancer cell growth *in vitro* was not observed in adult mice in comparison with young mice bearing a COLO320DM tumor, as shown by the bioluminescent signal (Figure 3B). Furthermore, no difference in invasion was observed as H&E staining revealed a non-invasive phenotype with pushing borders for both tumors from young and adult mice (Supplementary Figure S3). Therefore CAFs and their age may have an influence on CRC cells, but it is not likely to be the only factor responsible for rectal prolapse.

In conclusion, the phenotype of prolapse may be due to a combination of the anatomy of young mice, the non-invasive type of tumor, the serotonin secreted by the cancer cells and the young environment.

## MATERIALS AND METHODS

### Cell lines and cell culture

The human CRC cell line COLO320DM was isolated from a carcinoma of the sigmoid colon from a 55-year-old Caucasian female by Quinn et al. [14]. Double minutes (DM) were initially present in nearly all of the metaphases [14]. Cells grow both attached to the culture flask and in suspension. COLO320DM was cultured in glutamax enriched Roswell Park Memorial Institute medium (RPMI) 1640 supplemented with 10% fetal bovine serum (FBS) and antibiotics (100 U/ml penicillin, 100 μg/ml streptomycin). Cultures were maintained at 37°C in 5% CO_2_ in air. COLO320DM was chemically transfected with a firefly luciferase vector (pGL4.50 [luc2/CMV/Hygro] vector; Promega, Leiden, The Netherlands) by using TurboFect Transfection Reagent (Thermo Scientific, Erembodegem – Aalst, Belgium) following the manufacturer's recommendations. The human CRC cell line HT29 transfected with a firefly luciferase vector was kindly provided by Dr. Hackl (University of Toronto) [6]. HT29, HCT8/E11 and telomerase-immortalized human colon CAFs were cultured in Dulbecco's minimal essential medium (DMEM) supplemented with 10% FBS, 100 U/ml penicillin, 100 μg/ml streptomycin and 2 μg/ml fungizone (Life Technologies, Ghent, Belgium). Isolation and characterization of the CAFs was previously described [49]. Cultures were maintained at 37°C in 10% CO_2_ in air. All cultures were regularly tested for *Mycoplasma*. Phase/contrast microscope Leica DMI 3000B connected with Leica DFC 340FX and LAS4.1 software was used to make phase/contrast pictures. Authentication of both COLO320DM and HT29 was verified by short tandem repeat DNA profiling.

### Cell lysates and western blot

COLO320DM, luciferase transfected COLO320DM, HCT8/E11, CAFs and BLM transfected to overexpress P-cadherin were harvested in Laemmli lysis buffer (0.125 M Tris-HCl, 10% glycerol, 2.3% sodium dodecyl sulfate (SDS), pH 6.8). Cell lysates were suspended in reducing sample buffer (1 M Tris-HCl, 30% glycerol, 6% SDS, 3% β-mercaptoethanol, 0.005% bromophenol blue, pH 6.8) and boiled for 5 minutes at 95°C. Twenty μg proteins of each cell line were exposed to SDS-PAGE gels, transferred to nitrocellulose membranes (Bio-Rad, Hercules, CA, USA), blocked in 5% non-fat milk in phosphate-buffered saline (PBS) with 0.5% Tween-20 (Sigma-Aldrich, Belgium) and immunostained.

The following antibodies were used: mouse anti-CD29 (clone 18/CD29), mouse anti-P-cadherin (Clone 56/P-cadherin) (BD Biosciences), mouse monoclonal anti-Pan cadherin, monoclonal anti-β-catenin (Clone 15B8), mouse monoclonal anti-vimentin, mouse monoclonal anti-cytokeratin, pan antibody (Sigma-Aldrich, Belgium) and mouse monoclonal anti-human E-cadherin (clone HECD-1; Takara, Japan). The primary antibody against tubulin used for loading control was the mouse monoclonal anti-α-tubulin (clone B-5-1-2, Sigma-Aldrich, Belgium).

### Collagen invasion assay

Collagen invasion assay was performed as described previously [50]. Briefly, 1×10^5^ COLO320DM cells were seeded on 0.1% type I collagen gels (Santa Cruz, Santa Cruz, California, USA) in RPMI 1640 with 0.5% FBS. As a positive control, HCT8/E11 cells were seeded on 0.1% type I collagen gels in normal cell culture conditions. Morphology and collagen invasion were analyzed after 24h.

### xCELLigence Real-Time Cell Analysis (RTCA): migration

Real-time monitoring of cell migration was performed by using xCELLigence DP Real Time Cell Analyzer (RTCA, Westburg, Leusden, The Netherlands) according to the manufacturer's guidelines and as described by Limame et al [51]. Modified 16-well plates (Westburg) were used with upper and lower chambers separated by a microporous membrane containing 8 μm-pores. Microelectrodes are attached to the bottom of the membrane for impedance-based detection of migrated cells. In the lower chambers 160 μl medium with 10% FBS was added. In the upper well 50 μl of serum-free medium was added, followed by one hour incubation at 37°C and 5% CO_2_. Subsequently the background signal was measured. 160000 COLO320DM or HCT8/E11 cells were seeded per upper chamber in 100 μl serum-free medium. Each condition was performed in duplicate and impedance was measured each fifteen minutes for 24h. All data have been recorded by the supplied RTCA software 2.0. Migration was expressed as the cell index, i.e. the change in electrical impedance at each time point.

### Animals

Female immune-deficient Swiss nu/nu mice (Charles River Laboratories, l'Arbresle Cedex, France) were used. Animal studies were approved by the Animal Ethics Committee of Ghent University, Belgium.

### SC implantation of CRC cells

Six-weeks-old, which is considered as young, mice were inoculated SC with 1×10^6^ luciferase transfected COLO320DM cells alone (n=5) or in combination with 2.5×10^6^ non-irradiated (n=5) or irradiated (n=5) CAFs, in 50 μl PBS (Figure 1B). *In vitro* cultures of CAFs were exposed to 1.8 Gy daily for two weeks (five days/week) before inoculation, but no difference in results was obtained in mice inoculated with irradiated or non-irradiated CAFs.

### Orthotopic (intra-caecal) injection of CRC cells

Surgical procedures were performed under general anesthesia (IsoFlo, Abbott, Belgium) and analgesia (Ketoprofen, 5 mg/kg). A small midline laparotomy was executed and the caecum was located in the abdominal cavity. The caecum was then gently exteriorized and placed on a microscope slide connected to two bearers serving as a small table to stabilize the caecum and avoid pressure on the animal. The caecum was flattened with a forceps and by using a 300 μL insulin syringe with a 30G needle, a volume of 10 μL cells in serum-free DMEM was injected into the caecal wall under 2.5x magnification (OPMI 6, Zeiss, Germany). Successful injection was determined as the appearance of a small bleb without any leakage (Figure 1C). The caecum was then carefully returned to the abdominal cavity and the laparotomy was closed in two layers by sutures of PDS 6/0.

In a first group, adult mice, more than 12 weeks of age, were intra-caecally injected with 1×10^6^ luciferase transfected COLO320DM cells in combination with 2.5×10^6^ CAFs (n=8). Furthermore, six-weeks-old, young, mice were intra-caecally injected with 1×10^6^ luciferase transfected COLO320DM cells in combination with 2.5×10^6^ CAFs whether irradiated (n=8) or not (n=8). Exposure of CAFs to daily doses of 1.8 Gy for two weeks (five days/week) did not influence the outcome in mice (data not shown). In a last group, six-weeks-old, young, mice were intra-caecally injected with 1×10^6^ luciferase transfected HT29 cells in combination with 2.5×10^6^ CAFs (n=8).

### *In vivo* monitoring

Tumor development was weekly assessed by bioluminescence imaging until six weeks after inoculation. Mice were intraperitoneally injected with 150 mg/kg body weight Luciferin (Caliper Life Sciences, Hopkinton, MA) ten minutes before bioluminescence imaging, which was carried out by using an IVIS Lumina II (Caliper Life Sciences).

### Necropsy and histology

Animals were sacrificed when signs of disease were observed. Necropsy was performed and organs were sampled for histological examination. Organs were fixed with buffered formalin for 24 hours and subsequently paraffin-embedded. Standard H&E staining was applied to 5-μm thick sections. Sections were analyzed with a Leica DM750 light microscope. Pictures were taken with Leica MC170HD camera connected to the microscope using LAS4.3 software.

### Immunohistochemistry

Standard immunohistochemistry was applied to 5-μm thick sections using specific antibodies against CD56 (DAKO, Heverlee, Belgium; clone 123C3, 1/100), against chromogranin (Menarini, Zaventem, Belgium; clone 5H7, 1/200) and against synaptophysin (Menarini; clone 27G12, 1/100). Immunohistochemistry was performed on the BOND-MAX (Leica, Wetzlar, Germany). Briefly, the immunohistochemical expression was visualized using the Bond Polymer Refine Detection kit (Menarini; kit DS9800) for CD56 and chromogranin and using the Bond Intense R Detection (Menarini) for synaptophysin. The sections were counterstained with haematoxylin.

### Statistical analysis

Statistical analysis was performed with IBM SPSS statistics 21. The end point to apply statistics was set on six weeks post-inoculation. Survival curves were evaluated using Kaplan-Meier survival and log rank test. Comparison of prolapse in several groups was carried out by Fischer exact test. *In vivo* bioluminescent signals were analyzed by linear regression mixed models after log10 transformation. Bioluminescent signals for one time point were analyzed by Mann-Whitney U test.

## SUPPLEMENTARY MATERIALS FIGURES AND TABLES


