# Supplementary material for: Age and cellular context influence rectal prolapse formation in mice with caecal wall colorectal cancer xenografts
Source: Oncotarget. 2016 Sep 28;7(46):75603–15. doi: 10.18632/oncotarget.12312 (PMC5342764; doi:10.18632/oncotarget.12312)
Supplement: Supplementary file 1 [file oncotarget-07-75603-s001.pdf]

Age and cellular context influence rectal prolapse formation in mice with caecal wall colorectal cancer xenografts

SUPPLEMENTARY MATERIALS AND METHODS

Cell culture

Primary human colon CAFs were obtained from 10 patients with colon adenocarcinoma, submitted to surgical resection for therapeutic purposes [1]. CAFs were maintained in DMEM (Life Technologies, Ghent, Belgium) supplemented with 10% FCS and antibiotics. Phase/contrast microscope Leica DMI 3000B connected with Leica DFC 340FX and LAS4.1 software was used to make phase/contrast pictures.

Antibodies and reagents

Primary antibodies targeting the following antigens were used: p21 cip (R&D systems, Abingdon, UK); p-H2AX and H2AX Upstate Biotechnology (Lake Placid, NY, USA). Secondary antibodies coupled to horse radish peroxidase were obtained from Amersham Biosciences (Freiburg, Germany). The β-galactosidase kit was obtained from Sigma (Sigma-Aldrich, Belgium) and the test was performed according to the manufacturer's guidelines.

Secretome from primary human CAFs

Primary human colon CAFs were cultured in 75 cm^2 culture flasks. Cultures of young and replicative aged CAFs were washed three times with serum-free DMEM and incubated for 48 hours at 37 °C and 10% CO_2 with 9 ml serum-free DMEM. The secretome containing soluble factors derived from young and aged CAFs was harvested, centrifuged at 200 g for 5 min at 4 °C and passed through a 0.2 µm filter. The secretome from young and replicative aged CAFs was normalized to an equal number of cells. Secretome was stored at −20°C.

Cell growth assay with the secretome from young and replicative aged CAFs

COLO320DM cells transfected with luciferase (1.75 x 10^5/well) were seeded in a 24-well plate. CRC cells were treated with the secretome from young or replicative aged CAFs 24 h later. After 48 h D-luciferin (150 µg/mL) was added to the medium and cell numbers were analyzed by bioluminescent signal detection by using an IVIS Lumina II (Caliper Life Sciences, Hopkinton, MA, USA).

REFERENCES

1. De Wever O, Nguyen QD, Van Hoorde L, Bracke M, Bruyneel E, Gespach C, Mareel M. Tenascin-C and SF/HGF produced by myofibroblasts *in vitro* provide convergent pro-invasive signals to human colon cancer cells through RhoA and Rac. FASEB journal : official publication of the Federation of American Societies for Experimental Biology. 2004; 18:1016-1018.

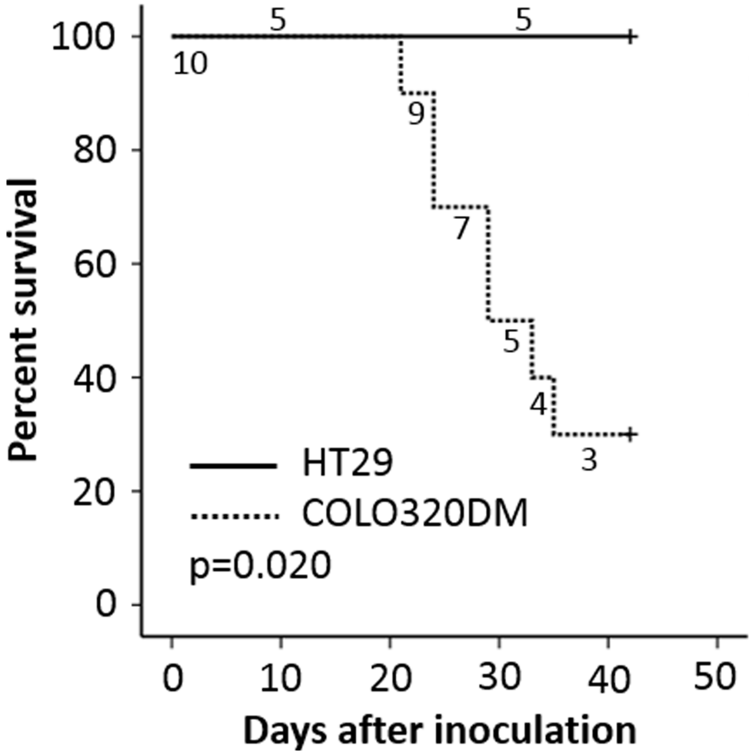

Supplementary Figure S1: Survival curve of young mice with a HT29 or COLO320DM caecum tumor.

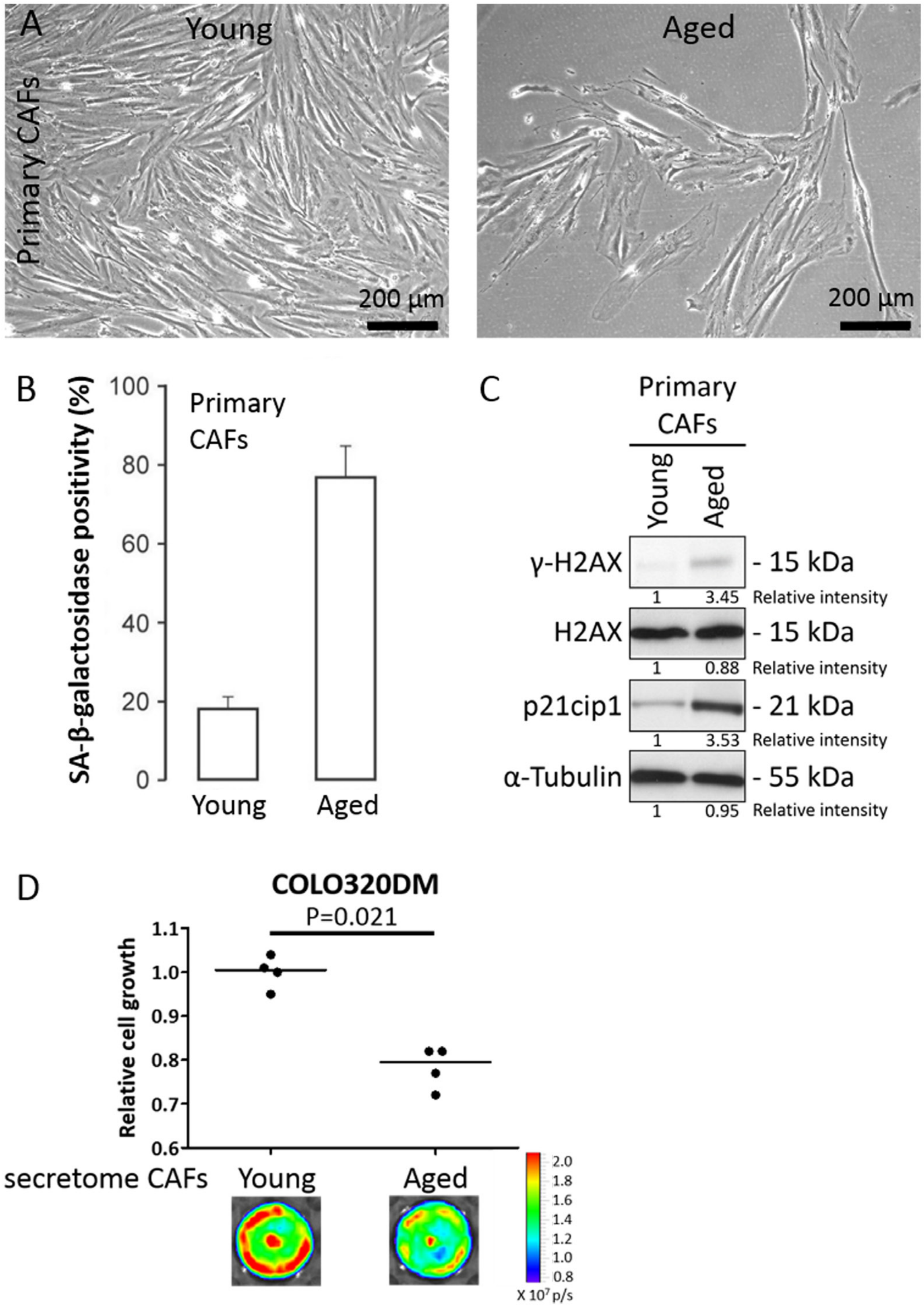

Supplementary Figure S2: Characterization of young and replicative aged human primary CAFs and impact on CRC cell growth. A. Phase/contrast pictures of young and replicative aged human primary CAFs. **B.** Quantification of senescence-associated β-galactosidase positivity. **C.** Western blot for different senescence markers in young and replicative aged human primary CAFs. **D.** Relative growth of COLO320DM cells transfected with luciferase. Quantification by bioluminescent imaging after 48 h treatment with the secretome of young or replicative aged human primary CAFs.

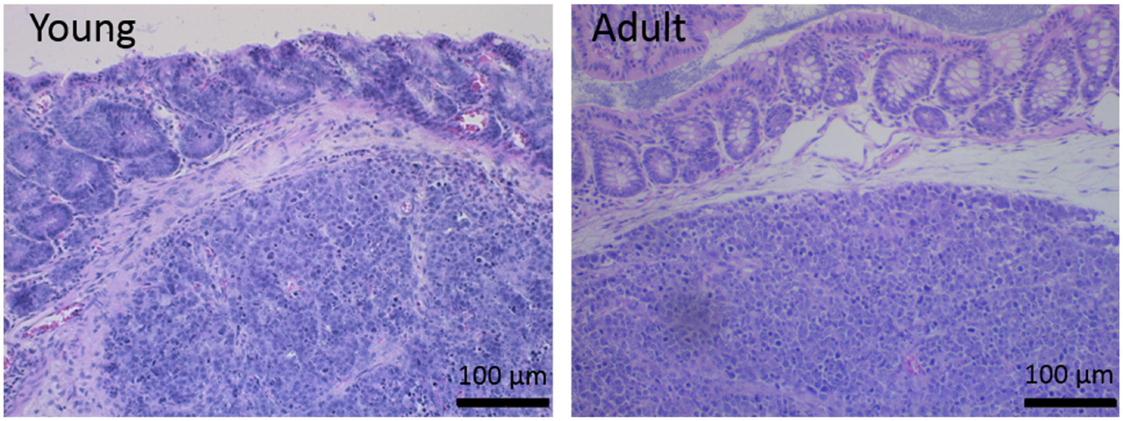

Supplementary Figure S3: Orthotopic COLO320DM tumors in young versus adult mice. H&E staining of the local COLO320DM tumor showing a non-invasive phenotype with pushing borders for both tumors from young and adult mice.
